# Linear Relationship between Resilience, Learning Approaches, and Coping Strategies to Predict Achievement in Undergraduate Students

**DOI:** 10.3389/fpsyg.2017.01039

**Published:** 2017-06-30

**Authors:** Jesús de la Fuente, María Fernández-Cabezas, Matilde Cambil, Manuel M. Vera, Maria Carmen González-Torres, Raquel Artuch-Garde

**Affiliations:** ^1^Department of Psychology, University of AlmeríaAlmería, Spain; ^2^Associate Researcher of Universidad Autónoma de ChileSantiago de Chile, Chile; ^3^Department of Developmental and Educational Psychology, University of GranadaGranada, Spain; ^4^Educational Psychologist, Ilustre Colegio Oficial de la Psicología de Andalucía OrientalGranada, Spain; ^5^María Inmaculada School, University of GranadaGranada, Spain; ^6^Department of Theory and Methods in Education and Psychology, School of Education and Psychology, University of NavarraPamplona, Spain; ^7^Department of Education and Psychology, Universidad Internacional de la RiojaLogroño, Spain

**Keywords:** resilience, learning approaches, coping strategies, academic performance, university stress

## Abstract

The aim of the present research was to analyze the linear relationship between resilience (meta-motivational variable), learning approaches (meta-cognitive variables), strategies for coping with academic stress (meta-emotional variable) and academic achievement, necessary in the context of university academic stress. A total of 656 students from a southern university in Spain completed different questionnaires: a resiliency scale, a coping strategies scale, and a study process questionnaire. Correlations and structural modeling were used for data analyses. There was a positive and significant linear association showing a relationship of association and prediction of resilience to the deep learning approach, and problem-centered coping strategies. In a complementary way, these variables positively and significantly predicted the academic achievement of university students. These results enabled a linear relationship of association and consistent and differential prediction to be established among the variables studied. Implications for future research are set out.

## Introduction

Previous studies have drawn attention to stress factors in academic contexts and their impact on mental health (Perfect et al., 2016). Moreover, although to a lesser extent, research on stress factors in the processes of academic learning has shown their relevance (Saklofske et al., 2012). This prior relevance is especially important if we bear in mind that the academic context is potentially stressful, depending on the combination of the characteristics of the student who is learning and of the characteristics of the teaching process (de la Fuente et al., 2017a).

*Academic stress* refers to the stressors and responses that occur in the academic field (Martin, 2007), which is why it is different from traumatic stress in persons of an academic age, since it refers to events of greater severity (Perfect et al., 2016). Some researchers have obtained results that have shown that university students experience higher stress levels when they start at university and in pre-exam periods (García-Ros et al., 2012) although these decline toward the end of the course. Final marks, homework, examinations and studying to overcome them can be considered as academic stressors (Misra and McKean, 2000). Carballo et al. (2011) reported the progressive changes in students' health habits, veering toward more harmful ones as they approached the examination period, with an increase in smoking, alcohol or psychoactive substances.

For these reasons, it is important to establish the relationship between the relevant psycho-educational variables and academic performance in the university contexts to determine the role of each to understand the response to academic stress. The purpose of this research was to establish the linear relationship between resilience, learning approaches, and coping strategies to predict achievement in undergraduate students in stressful academic university contexts based on the model presented below.

### Competence to learn to learn in stressful academic contexts

The competence to deal with academic stress is multidimensional. It refers to the set of knowledge, skills and meta-skills, attitudes, values and habits that a person has developed, which allows her or him not only to face specific situations of evaluation but to do so successfully, according to the model of *Competence of Learning, Studying, and Performing under Stress*, with the acronym CLSPS™ (de la Fuente, 2015) and to synthesizes different behavioral levels (See Chart 1):

1. *The level of conceptual sub-competence (to know)* about a subject or matter in the field of higher studies requires the student to know about the knowledge of *facts, concepts and principles* relating to the subject. *Specific knowledge* about the facts of a subject is fundamental to decide about it. The *knowledge of facts* about the time it takes to prepare it, the qualifications, the possibility of preparing it alone or in groups, or other aspects, is associated with adequate decision making at the time of beginning it and the time to be dedicated to it, the effort to carry it out, competitive possibilities, etc. Moreover, to study a subject one has to start from a set of *principles* relating to the subject. These principles are referred to a system of beliefs, standards or explanatory behavioral processes that are behavioral predictors of motivation, emotion and effort. If the principles are adjusted they will help to maintain effort and motivation, and if they are inadequate the student will abandon the good intentions of effort producing self-induced stress and anxiety. It is necessary to evaluate these types of beliefs or principles before beginning the course.
2. At the level of *procedural sub-competence* (*know-how*), the skills and cognitive meta-skills of studying and learning are associated with motivation during study and the performance obtained. When a student is motivated s/he tends to use a deep learning approach, while a surface one to learning applies in the opposite case. Research on the *deep approach* has established that it is associated with good learning and study strategies (Camarero et al., 2000). These skills are essential because they enhance the learning process and optimize the construction of the required knowledge. Therefore, before beginning to study a subject it is important to know if the skills of study and learning are appropriate. An essential task of a university student is to adjust studying and learning skills to the proposed assessment system.
3. *The level of attitudinal sub-competence* (*knowing how to know, want to know, to be*). It's refer to attitudes, values and habits. This knowledge is built, skills are practiced, but attitudes are internalized personally. In this way the student has to want to achieve something, want to fight for something, and be motivated to succeed at something. It has recently been shown that students can be helped to generate self-motivation strategies. The attitude toward learning and study is related to the set of thoughts, feelings and actions that make it probable that the conduct of effort and persistence is maintained to accomplish certain achievements.

**Chart 1:** Multidimensional nature of the Competency for Studying, Learning and Performing under Stress (CSLPS model; de la Fuente, 2015).

1) To know: (Knowledge)	*Facts*: knowledge about the characteristics of the class subject or professional exam: job openings, percentage of candidates who pass, requirements.
	*Concepts*: competitive exam system, requirements; type of examination, scoring, prior merits/credits, type of class subject.
	*Principles*: beliefs about the professional exam or selection process.
2) Know how: (Skills)	*Instrumental skills*: written and oral skills.
	*Learning and study skills*: study skills and techniques.
	*Meta-cognitive skills for study*: **learning approaches**.^*^
	*Meta-emotional skills for managing stress*: **coping strategies**.^*^
	*Meta-motivational skills for managing stress*: **resilience**.^*^
	*Meta-behavioral skills for managing stress*: self-regulation strategies.
3) Know to be: (Attitudes)	*Attitudes and values*: behavioral confidence, achievement motivation, mindset.
	*Study habits* (time management, persistence, discipline).

* *Variables in this research*.

### Resilience as a meta-motivational variable

Resilience is a personal variable that recent research has shown to be very relevant (Martin, 2002, 2013; Martin and Marsh, 2006, 2008; Artuch-Garde, 2014; Edwards et al., 2016; Artuch-Garde et al., 2017). The definition of Bermejo (2011) can be taken as capacity, the result of the interaction of different personal variables with environmental factors, which allows the individual to confront and solve, in an adequate and integrated manner in their cultural environment, different situations of adversity, risk, or trauma for different reasons, allowing it to reach a normalized situation and adapted to its cultural environment (p.8).

In the academic field, resilience plays a significant role as a *motivational-affective* variable, so that, in addition to being a stimulus for the realization of academic and personal goals, it provides adequate mechanisms to deal with adverse situations of stress and anxiety that arise in the university environment (Fernández-Castillo and Gutiérrez, 2009; Allan et al., 2014; González-Torres and Artuch-Garde, 2014; Cassidy, 2015). The student measures his or her own forces in the face of different challenges and demands, not only academic but also psychosocial, negotiating demanding situations that lead to moments in which he must confront himself in order to better understand his potential and abilities, to learn and respond efficiently, retaining his mental health and confidence in his potential and abilities. For this reason, it has been considered as a *meta-motivational* variable, regulating one's own motivation (de la Fuente, 2015).

The learning process involves a great deal of motivation, which means not only adequately resisting rhythms, adaptive demands and responses of all kinds, but also having the ability to self-motivate to respond in the right way without falling into situations of exacerbation or emotional distress, such as helplessness, apathy, depression or distress (Alvarez-Ramírez and Cáceres, 2010). Some previous studies about resilience in student populations reveal these manifestations associated with a deficiency of resilience (Bragagnolo et al., 2005). Likewise, stress research in university students indicates that a lack of self-confidence creates a vulnerability that leaves students in conditions of scant resistance and little optimism about their possibilities and those of the environment to be able to get ahead, which triggers diverse psychosocial problems (Solórzano and Ramos, 2006).

### Learning approaches as a meta-cognitive variable

Meta-cognition has two distinctive characteristics: the knowledge of knowledge and the control of cognitive processes. It also includes knowledge about personal characteristics such as skills, abilities and experiences, as well as knowledge of strategies that can be used to address the task. The competent learner undertakes the processes of control that are directed to the organization and planning of the cognitive activity toward a goal. In addition it enables the student to direct, regulate and supervise the course of cognitive activity and to evaluate the follow-up together with the results obtained according to the established goal (Pintrich, 1999, 2002).

Biggs (1988) defined learning approaches as learning processes that emerge from students' perceptions of academic tasks influenced by their personal characteristics. They are characterized for the student's intention or motive and the learning strategy used for the study (Barros et al., 2013). He points out that in learning approaches there are two different levels of study (Biggs, 1993, 2001): one, more precisely directed to a specific task (approach as process) and the other, more general (approach as predisposition). The motives and strategies that make up learning approaches are *deep motivation* (the interest or motivation is intrinsic to the task), *surface motivation* (extrinsic to the purpose of the task, with which the student learns to avoid failure with the least possible effort), deep *strategy* (the strategies needed to achieve the understanding of the task and its meaning) and the *surface strategy* (reproduction of the material through repetition, which are strategies focused on responding to the demands of the evaluation. For all this, the model considers them with a meta-cognitive order.

### Coping strategies as a meta-emotional variable

Lazarus and Folkman (1984) and Folkman and Moscowitz (2004) defined coping as constantly changing cognitive and behavioral processes that are developed to handle specific external and / or internal demands that are valued as beyond the individual's resources. These responses allow us to manage and reduce, in some way, the adverse qualities of a stressful situation, thus serving as an attempt to manage stressors. Coping *styles* refer to predispositions of personality that transcend the influence of the situational and temporal context (Felipe and León, 2010). It emphasizes the stability of coping in different situations, rather than the change in the use of strategies (Carver and Connor-Smith, 2010). On the other hand, coping *strategies* (or responses) are particular thoughts and behaviors carried out in response to stressful situations that may change over time, are contextual and can be changing depending on the triggering conditions (Piemontesi et al., 2012).

Research on coping strategies (Ticona et al., 2010; Hung, 2011) seems to show agreement on three types of strategies: (a) Problem-focused coping strategies; (b) coping strategies centered on emotion, and (c) avoidance coping strategies of abandonment of control or escape responses. *Strategies directed at the problem* aim to solve the problem. Planning, instrumental coping, the search for support and information and confrontation are of this type and usually manage to reduce the emotional malaise. However, their effects can be counterproductive in situations that cannot be changed or uncontrollable problems. Strategies aimed at *emotional regulation*, including cognitive-behavioral avoidance, abandonment, affective discharge, talking about emotions and repeatedly thinking about the problem usually result in not improving or even worsening the affective discomfort. Strategies that seek to give meaning to what happened, positive reassessment of one self, the world and the social context, the search for emotional social support would have positive effects. However, repeated thinking, or attributing to oneself all responsibility for failure would have negative effects, if they occur in a maladjusted way.

The CLSPS model has established that coping strategies are procedural variables that function by operating as a *meta-emotional variable*, since it defines them as emotional behavioral management skills to cope with the stress that study and learning in university situations potentially leads to. They are fundamental since they enable emotional regulation during all the time that the study phase lasts (Chou et al., 2011) or on the contrary in this case, carry associated health problems (Sulkowski et al., 2011).

### Academic performance

The teaching-learning process is directed toward a particular product. In order to achieve this product, it is necessary beforehand to start from some objectives and aims which it is fundamental that the student learns. This product that is obtained from the teaching-learning process is what is called academic performance. Academic performance is a key factor in higher education, since it constitutes one of the most powerful variables in the teaching-learning process. Much research has been done globally on academic performance, although this tendency to reduce learning outcomes to a single end has been criticized. de la Fuente et al. (2008) define academic performance as a composite of learning results in three spheres: conceptual, procedural and attitudinal. Thus we have a global performance that can be broken down into its three subcomponents: conceptual (grades obtained on exams), procedural (class attendance and lab work) and attitudinal (class participation and voluntary efforts).

### Objectives and hypothesis

These are based on the contextual framework of the CLSPS™ model that seeks to establish the linear relationships of association and prediction among the variables reviewed (meta-meta-motivational, meta-emotional and meta-cognitive), as well as their overall predictive value and differentials of academic university performance. Therefore, the *objectives* are related to the questions: (1) What is the relationship of association between all the variables (resilience, coping strategies and learning approaches)? (2) Is a linear predictive empirical model that establishes the structural linear relationships between the constructs studied to predict academic performance possible? To this end, the following *hypotheses* were established:

#### Association hypothesis

1. *Total resilience and its components* were expected to be significantly associated, in a linear and positive fashion, with the *deep approach* - with special emphasis on *deep motivation* - and negatively on the *surface approach* - similarly with surface motivation.
2. Total resilience and its components were expected to be positively associated with *problem-focused* coping strategies and negatively with *emotion-focused* coping strategies.
3. It was expected that the *surface approach* would be associated, in a positive linear manner, with *emotion-focused coping strategies*. It was expected that the *deep approach* would not be associated with *emotion-focused* coping strategies, given the low level of stress experienced by these types of students.

#### Hypothesis of lineal structural prediction

(4) It was expected that the constituent components of *resilience* would have a positive significant prediction of the deep approach and negative of the surface approach, as well as a positive predictor of *strategies centerd in the problem* and negatives on those *centerd in the emotions*. Furthermore, that *resilience* would have a positive and predictive linear relationship, together with the *deep approach* and coping *focussed on the problems of academic performance*.

## Methods

### Participants

A total of 656 students from a University of the south of Spain participated in this study, with a mean age of 22.55 (*SD* = 3.78) years. The percentage of men in this study was 21.2% (*n* = 246) while that of women was 78.8% (*n* = 410). These university students are enrolled in undergraduate and graduate degrees in Psychology, attending second (*n* = 260) and fourth years (*n* = 396). It is important to note that the students did not complete all the questionnaires equally. Thus, only 312 complied with resilience, 447 with coping strategies, and 476 with learning approaches.

### Instruments

#### Meta-motivational evaluation

##### Connor-Davison resilience scale

CD-RISC (Connor and Davidson, 2003) was used in a Spanish validated version (Mateu et al., 2010; Notario-Pacheco et al., 2014). This Likert type scale contains 25 items and five factors: (1) personal competence, high standards and tenacity (0.80), (2) self-confidence, tolerance of negative affect and strengthening effects of stress (0.75), (3) Positive acceptance of change, and secure relationships (0.77), (4) control (0.71), and (5) spiritual influences (0.61).

#### Meta-cognitive evaluation

##### Learning approaches

The Revised Two-Factor Study Process Questionnaire, R-SPQ-2F (Biggs et al., 2001), in its validated Spanish version (Justicia et al., 2008) was used to identify the different learning approaches that predominate in our university students. The learning approaches variable is composed of four subscales, deep motivation, deep strategies, surface strategy, and surface motivation, giving rise to the dimensions of deep approach and surface approach, respectively. It is composed of 20 items on a Likert scale from 1 (Never or rarely) to 5 (Always or almost always). The questionnaire also possesses adequate validity and reliability values. It contains four subscales: motivation and deep strategy (0.83 and 0.89 respectively), motivation and surface strategy (0.81 and 0.86, respectively).

#### Meta-emotional evaluation

*The Coping Strategies Scale, EEC* (Chorot and Sandín, 1987) was used in the *Short EEC Scale* (de la Fuente, 2014). Although the original instrument contained 90 items, the validation produced a first-order structure of 64 items and a second order with 10 factors and two dimensions, both of them significant, with adequate fit values in the latter case (Chi-square = 878.750; Degrees of freedom (77–34) = 43, *p* < 0.001; NFI = 0.901; RFI = 0.945; IFI = 0.903, TLI = 0.951, CFI = 0.903). Reliability measures are Cronbach alpha of 0.93 (complete scale), 0.93 (first half) and 0.90 (second half), Spearman-Brown of 0.84 and Guttman of 0.80. It evaluates two dimensions, (D1) problem-centered coping (0.91) and (D2) emotion-focused coping (0.95). In relation to *emotion-focused* strategies these were: Evasive distraction (0.79), Reduction of anxiety and avoidance (0.88), Preparing for the worst (0.80), Emotional shock and isolation (0.91) and Resigned acceptance (0.86). In relation to *problem-centerd* strategies: Search for family counseling and help (0.92), Self-instruction (0.82), Positive reassessment, and firmness (0.87), Communication of feelings and social support (0.89), and Search for alternative reinforcements (0.80). See Table 1.

**Table 1:** Types of coping strategies and examples of items.

**Coping centered in emotion (D2)**	**Example of items**
F1. Evasive distraction	I get away and forget the problem temporarily (change of environment)
F7. Reduction of anxiety and avoidance	I practice some kind of sport in order to reduce my anxiety or tension
F8. Preparing for the worst	I prepare myself for the worst
F9. Emotional discharge and isolation	I act irritable and aggressive toward others
F11. Resigned acceptance	I accept the problem as it is, since I cannot do anything to solve it
**Coping centered on the problem (D1)**	
F2. Search for help and family advice	I talk with people I know who can do something to solve my problem
F5. Self-Instruction	I set down a plan of action and try to carry it out
F10. Positive Re-evaluation and firmness	I try to see positive aspects of the situation
F12. Comunication of feelings and social support	I feel better if I explain my problem to friends or family members
F13. Search for alternative reinforcement	I start new activities (studies, etc.)

#### Academic performance

The scores of the subjects studied were obtained and provided by the tutor teachers. Out of a total of 10 points, *conceptual* learning (4 p) came from a 40-question test, *procedural* learning (4 p) from the practical activities performed, and the *attitude* learning (2p) from the complementary activities of participation.

### Procedure

Participants in this research received the same information, which was provided through the *Academic Stress e-Coping platform* (de la Fuente et al., 2015) in the context of a more extensive research developed within the R & D Project ref. (2012–2015). It is available in web format: http://www.estres.investigacion-psicopedagogica.com/english/seccion.php?idseccion=7. The evaluation was carried out in the subject of Psychology of Education, given in the degrees of Bachelor (4th year, 2012–2013) and Degree of Psychology (2nd year, 2014–2015), with the same methodology and teacher to minimize external contaminating factors. Participating students completed the questionnaires voluntarily during the class hours of both courses, coinciding with the first and second semester, respectively. The collection and processing of the data were done voluntarily, with the informed consent of the students, accepting the Ethical and Deontological Principles of Psychology. The data were processed in an anonymous and group format, being protected in a database guarded at the University. The Bioethics Committee approved the Project and the instruments.

### Data analysis

An ex-post-facto prospective design was used, manipulating the independent variables by selection. For the *association* hypotheses Pearson's bivariate correlation coefficients were used, as well as structural equations model (SEM) for *structural* analyses. The analyses of correlation provided knowledge of the bivariate relationship between the direct variables, while the pathway analyses provided knowledge of simultaneous predictions taking into consideration the direct and indirect effects among the latent variables defined. Statistical programmes SPSS (v. 22) and AMOS (v. 22) were used with Licence for use in the Universities of Almería and Granada, (Spain).

## Results

### Bivariate association between resilience and learning approaches

Regarding the *factors of resilience*, in the *tenacity* factor, significant positive relations appeared with the factors of the deep approach, whereas they were negative with the constitutive factors of the surface approach such as the surface strategy (*r* = −175, *p* < 0.01), surface motivation (*r* = −0.120, *p* < 0.05) and surface approach. The *stress tolerance factor* also appeared positively and significantly associated with the deep approach (*r* = 0.229; *p* < 0.01) and its components, but not with the surface approach, as well as the positive correlation control factor (*r* = 0.217, *p* < 0.01), deep strategy (*r* = 0.162; *p* < 0.01) and with deep motivation (*r* = 0.238; *p* < 0.01). See Table 2.

**Table 2:** Bivariate correlations between Resilience and Learning Approaches.

	**Tenacity**	**Stress**	**Change**	**Control**	**Spirituality**	**Total resilience**
Deep strategy	0.346^**^	0.192^**^	0.094	0.162^**^	0.003	0.195^**^
Deep motivation	0.322^**^	0.229^**^	0.162^**^	0.238^**^	0.091	0.322^**^
Deep approach	0.365^**^	0.219^**^	0.138^*^	0.217^**^	0.050	0.279^**^
Surface strategy	−0.175^**^	−0.040	−0.114	−0.092	0.036	−0.131^*^
Surface motivation	−0.120^*^	0.085	−0.086	−0.104	0.039	−0.094
Surface approach	−0.165^**^	0.023	−0.111	−0.108	0.041	−0.125^*^

* *p < 0.05*,

** *p < 0.01*.

### Bivariate association between resilience and coping strategies

The total resilience score correlated significantly and positively with the *problem-centered coping strategy* (*r* = 0.121; *p* < 0.05), but not with that focused on emotion. Only the *spirituality* factor appeared with a significant positive correlation with the total result of the coping strategies (*r* = 0.186; *p* < 0.01). There were also significant positive relationships between the *control* of the resilience component and *problem-focused coping strategies* (*r* = 0.158; *p* < 0.05) and significant negatives with emotion-focused strategies (*r* = −0.158; *p* < 0.05). In addition, significant positive relationships between the *spirituality* factor and problem-centered strategies also appeared (*r* = 0.145; *p* < 0.05). See Table 3.

**Table 3:** Bivariate correlations between resilience and coping strategies.

	**Tenacity**	**Stress**	**Change**	**Control**	**Spirituality**	**Total resilience**
Coping strategy emotion	−0.018	0.046	−0.046	−0.158^*^	0.161	0.014
Coping strategy problem	0.035	−0.045	0.103	0.158^*^	0.145^*^	0.121^*^
Total coping	−0.001	−0.019	0.047	0.016	0.186^**^	0.084

* *p < 0.05*,

** *p < 0.01*.

The association relationships between *total resilience* and *coping factors* showed that very significant and positive correlations were maintained with each of the factors of coping strategies focused on the problem. However, in the case of emotion-focused coping strategies, a significant negative correlation with *emotional shock* and *isolation* was observed (*r* = −0.225, *p* < 0.01).

In the association between the *factors of resilience* and the factors of *emotion-centered* coping strategies, it is important to note the significant negative correlations of the perception of *change management* (*r* = −0.180, *p* < 0.01) and of *control* (*r* = −0.205; *p* < 0.01), with *emotional discharge* and *isolation*. Another significant negative correlation to be highlighted is the relationship between the perception of *control* and *resigned acceptance* (*r* = −0.203; *p* < 0.01) and *preparing for the worst* (*r* = −0.147; *p* < 0.05). Less significant but of interest is the positive relationship between *stress management* and *evasive distraction* (*r* = 0.142; *p* < 0.05) and stress (positively) and between *control and preparation for the worst* (in this case negatively; *r* = −147; *p* < 0.05).

In the association between *resilience factors* and factors of *problem-focused coping strategies*, it can be seen that all factors of *resilience* showed significant positive relationships with *self-instruction* strategies (e.g., in stress, *r* = 0.310; *p* < 0.01) and *positive reassessment and firmness* (e.g., tenacity, *r* = 0.439; *p* < 01), and less with the spiritual factor. There are other significant correlations between the factors of *change* and *control* with the search for help and family counseling, and specifically the *control* factor with the *communication of feelings and the search for social support*. See Table 4.

**Table 4:** Bivariate correlations between Resilience factors and coping Strategies, ordered by dimensions and factors.

	**Tenacity**	**Stress**	**Change**	**Control**	**Spirituality**	**Total resilience**
**STRATEGIES CENTERED EMOTION (D2)**						
F1. Evasive distraction	0.055	0.142^*^	0.048	−0.076	0.206^*^	0.129^*^
F7. Reduction of anxiety and avoidance	−0.073	−0.004	0.002	−0.090	0.064	0.001
F8. Preparing for the worst	−0.037	0.045	−0.008	−0.147^*^	0.025	−0.013
F9. Emotional discharge and isolation	−0.143^*^	−0.065	−180^**^	−0.205^**^	−0.090	−0.225^**^
F11. Resigned Acceptance	−0.086	−0.001	−0.106	−0.230^**^	0.025	−0.099
**STRATEGIES CENTERED PROBLEM (D1)**						
F2. Search for help and family advice	0.074	−0.019	0.161^**^	0.233^**^	0.110	0.168^**^
F5. Self-Instruction	0.293^**^	0.310^**^	0.307^**^	0.227^**^	0.087	0.366^**^
F10. Positive re-evaluation and firmness	0.439^**^	0.466^**^	0.382^**^	0.230^**^	0.023	0.434^**^
F12. Communication of feelings and social support	0.011	−0.103	0.108	0.286^**^	0.173^**^	0.175^**^
F13. Search for alternative reinforcement	0.088	0.136^*^	0.164^**^	−112	0.098	0.162^**^

* *p < 0.05*,

** *p < 0.01*.

### Bivariate association between learning approaches and coping strategies

In this case, only a *significant negative relationship* was found between *deep motivation* and *emotion-focused* coping strategies (*r* = −0.118; *p* < 0.05). One striking issue is that both approaches, strategies and deep motivation, have a negative relationship with both coping strategies. See Table 5.

**Table 5:** Relation between the variables of learning approach with coping strategies.

	**Deep strategy**	**Deep motivation**	**Deep approach**	**Surface strategy**	**Surface motivation**	**Surface approach**
Coping emotion	−0.053	−0.118^*^	−0.093	0.018	0.017	0.019
Coping problem	−0.014	−0.058	−0.039	−0.072	−0.078	−0.082
Total coping	−0.042	−0.101	−0.077	−0.019	−0.025	−0.024

* *p < 0.05*,

** *p < 0.01*.

In the analysis by factors, it appeared that *deep approach* and *deep motivation* have a significant negative relation with all of the strategies *focused on the emotion*, whereas there is no relationship of the *deep strategies* with those strategies. Neither the *surface approach* nor its components with strategies *focused on emotion*. In the case of the relationship between *learning approaches* and *problem-focused coping strategies*, the relationship is linear and inversely significant with two specific strategies. Thus, the *deep approach and its components* positively and significantly correlated with *self-instruction*, and *positive reevaluation strategies*, while the *surface approach and its components* were negatively and significantly associated to both strategies. In addition, the surface approach and surface motivation appeared associated negatively to the *communication of feelings and social support*. See Table 6.

**Table 6:** Bivariate correlation between the variables of learning approach with coping strategies by dimensions and factors.

**Coping Strategies**	**Deep strat**	**Deep mot**	**Deep appr**	**Surface strat**	**Surface mot**	**Surface appr**
**CENTERED ON THE EMOTIONS (D2)**						
F1. Evasive distraction	−0.087	−0.100^*^	−0.102^*^	−077	0.045	0.068
F7. Reduction of anxiety and avoidance	−0.071	−0.113^*^	−0.100^*^	0.061	0.027	0.049
F8. Preparing for the worst	−0.094	−0.136^**^	−0.125^*^	0.004	0.052	0.030
F9. Emotional discharge and isolation	−0.094	−0.142^**^	−0.129^*^	0.056	0.046	0.056
F11. Resigned acceptance	−0.084	−0.143^**^	−0.124^*^	0.024	0.034	0.032
**CENTERED ON THE PROBLEM (D1)**						
F2. Search for help and family advice	0.033	0.014	0.026	−0.098	−0.081	−0.099
F5. Self-Instruction	0.147^**^	0.148^**^	0.162^**^	−0.258^**^	−0.222^**^	−0.264^**^
F10. Positive Re-evaluation and firmness	0.177^**^	0.180^**^	0.197^**^	−0.199^**^	−0.125^*^	−0.180^**^
F12. Comunication of feelings and social support	−0.005	0.002	−0.002	−0.085	−0.104^*^	−0.103^*^
F13. Search for alternative reinforcement	0.009	−0.029	−0.010	−0.073	−0.078	−0.083

* *p < 0.05*,

** *p < 0.01*.

### Structural analysis

The results of structural analysis or pathway analysis (SEM) showed an acceptable model of relationships between variables. The relationship parameters of both models are set out below. See Table 7.

**Table 7:** Models of structural lineal results of the variables.

**Chi^2^**	**FG**	***p* <**	**NFI**	**RFI**	**IFI**	**TLI**	**CFI**	**HOELTER**	**RMSEA**
Model 1470,065	(252-75):177	0.000	0.868	0.830	0.880	0.847	0.883	0.184	0.078
Model 1546,762	(275–74):201	0.000	0.910	0.923	0.914	0.921	0.914	0.200	0.072

#### Standardized direct effects

This predictive linear model establishes that latent variable *resilience* (D1) was a significant predictor (0.32) of the latent variable *deep approach* (D2). In addition, it positively predicted (0.52) of latent variable *problem-centered strategies* and negatively predicted (−0.27) for latent variable *emotion-focused strategies*. At the same time, the *deep learning approach* (D2) was a negative predictor (−0.24) of *problem-centered* strategies (D4).

Moreover, there appeared a significant and negative (−0.38) predictive relationship among the latent variables *deep approach* (D2) and *surface approach* (D3). *C*omplementarily, the latent variable *deep approach* predicted negatively and significantly a emotion-focused coping strategy (D4), while the *surface approach* (D3) significantly and positively predicted (0.16) a problem -*focused strategy* (D5). Finally, the problem-*centered* coping strategy (D4), also significantly and positively predicted (0.95) *emotion-focused strategies* (D5), as well as the latent variable academic performance (0.25). All the variance of errors were significant (*p* < 0.001). Table 8 shows the *direct effects* of the variables inherent in the model.

**Table 8:** Standardized direct effects (Default model).

	**D1**	**D2**	**D3**	**D4**	**D5**	**D6**
D2	0.322					
D3		−0.382				
D4	0.519	−0.239				
D5	−0.266		0.156	0.955		
D6				0.251		
TENACITY	0.665					
STRESS	0.631					
CHANGE	0.668					
CONTROL	0.708					
SPIRITUALITY	0.125					
DEEPSTR		0.715				
DEEPMOT		0.958				
SURFSTR			0.814			
SURFMOT			0.791			
CF13				0.895		
CF2				0.792		
CF10				0.795		
CF5				0.837		
CF12				0.720		
CF8					0.882	
CF7					0.879	
CF1					0.886	
CF11					0.878	
CF9					0.905	
CONCEP						0.394
PROCED						−0.747
ATTITUD						−0.947

*D1, Resilience; D2, Deep approach; D3, Surface approach; D4, Coping emotion; D5, Coping problem; D6, Academic Achievement; TENACITY, Tenacity; ESTRES, Tolerance to stress; CHANGE: Change; CONTROL, Perception of control; SPIRITUALITY, Spirituality; CF13, Search for alternative reinforcement; CF2, Search for help and family advice; CF10, Positive re-evaluation and firmness; CF5, Self-instructions; CF12, Communication of feelings and social support; CF8, Preparing for the worst; CF7, Reduction of anxiety and avoidance; CF1, Evasive distraction; CF11, Resigned acceptance; CF9, Emotional discharge and isolation; DEEPSTR, Deep strategy; DEEPMOT, Deep motivation; SURFSTR, Surface strategies; SURFMOT, Surface motivation; CONCEPT, Conceptual achievement; PROCED, Procedural achievement; ATT, Attitudinal achievement*.

#### Standardized indirect effects

The model also contributed the existence of *multiple indirect* predictions among the variables. This predictive linear model establishes that latent variable resilience (D1) was a negative significant predictor (−0.123) of the latent variable *surface approach* (D3), negative predictor of latent variable *coping-focused emotion* (−0.077), positive predictor (0.403) of *emotion coping*, and positive predictor (0.111) of *achievement* (D6). In addition, the latent variable *deep approach learning* was negative predicted (−0.288) of latent variable *emotion-centered strategies* (D5) and academic achievement (−0.060) (D6).

In addition, there was another indirect predictive and negative (−0.288) relationship between the latent variable *deep approach* (D2) and the *strategies focused on emotion* (D5). Complementarily, the latent variable *resilience* positively predicted the coping strategies of *problem-focused coping* (CF13, CF2, CF10, CF5, and CF12) and, less strongly, emotion (CF8, CF7, CF1, CF11, and CF9), while the *surface approach* did so negatively for both groups of types of *coping strategies*.

The latent variable of *resilience* (D1) also appeared with a positive indirect effect on the *deep approach* (D2) components (deep motivation = 0.230, deep strategy = 0.308) and with a negative effect on the *surface approach* (D3) (surface motivation = 0.097; surface strategy = 0.100) while the *deep approach* (D2) appeared with the negative predictive effect of the components of the *surface approach* (surface strategy = −0.311; surface motivation = −0.302). The latent variable *of the surface approach* (D3) appeared with a negative predictive value of the components of *problem-centered strategies* (CF8, CF7, CF11, and CF9), while problem-centered coping latent variable (D4) appeared as predictors of the components focused *in the emotion* (D5).

Finally, there was a differential effect with regard to the prediction of the types of academic performance. Thus, while the latent variable *resilience* (D1) had an indirect predictive positive effect for *conceptual* performance (0.044) it was a negative sign for *procedural* (−0.083) and *attitudinal* (−0.105) performance, the tendency was inverse for the latent variables *deep approach* (D2) and *problem-centered strategies* (D4). Table 9 shows the *indirect effects* commented on among the variables inherent in the model.

**Table 9:** Standardized indirect effects (Default Model).

	**D1**	**D2**	**D3**	**D4**	**D5**	**D6**
D2						
D3	−0.123					
D4	−0.077					
D5	0.403	−0.288				
D6	0.111	−0.060				
TENACITY						
STRESS						
CHANGE						
CONTROL						
SPIRITUALITY						
CF13	0.395	−0.214				
CF2	0.350	−0.189				
CF10	0.351	−0.190				
CF5	0.370	−0.200				
CF12	0.318	−0.172				
CF8	0.121	−0.254	0.138	0.842		
CF7	0.120	−0.253	0.138	0.840		
CF1	0.121	−0.255	0.139	0.846		
CF11	0.120	−0.253	0.137	0.838		
CF9	0.124	−0.260	0.142	0.864		
DEEPSTR	0.230					
DEEPMOT	0.308					
SURFSTR	−0.100	−0.311				
SURFMOT	−0.097	−0.302				
CONCEPTUAL	0.044	−0.024		0.099		
PROCEDURAL	−0.083	0.045		−0.187		
ATTITUDINAL	0.105	0.057		−0.238		

*D1, Resilience; D2, Deep approach; D3, Surface approach; D4, Coping emotion; D5, Coping problem; D6, Academic Achievement; TENACITY, Tenacity; ESTRES, Tolerance to stress; CHANGE, Change; CONTROL, Perception of control; SPIRITUALITY, Spirituality; CF13, Search for alternative reinforcement; CF2, Search for help and family advice familiar; CF10, Positive re-evaluation and firmness; CF5, Self-instructions; CF12, Communication of feelings and social support; CF8, Preparing for the worst; CF7, Reduction of anxiety and avoidance; CF1, Evasive distraction; CF11, Resigned acceptance; CF9, Emotional discharge and isolation; DEEPSTR, Deep strategy; DEEPMOT, Deep motivation; SURFSTR, Surface strategies; SURFMOT, Surface motivation; CONCEPT, Conceptual achievement; PROCED, Procedural achievement; ATT, Attitudinal achievement*.

#### Graphic representation of the structural model

**Figure 1:**
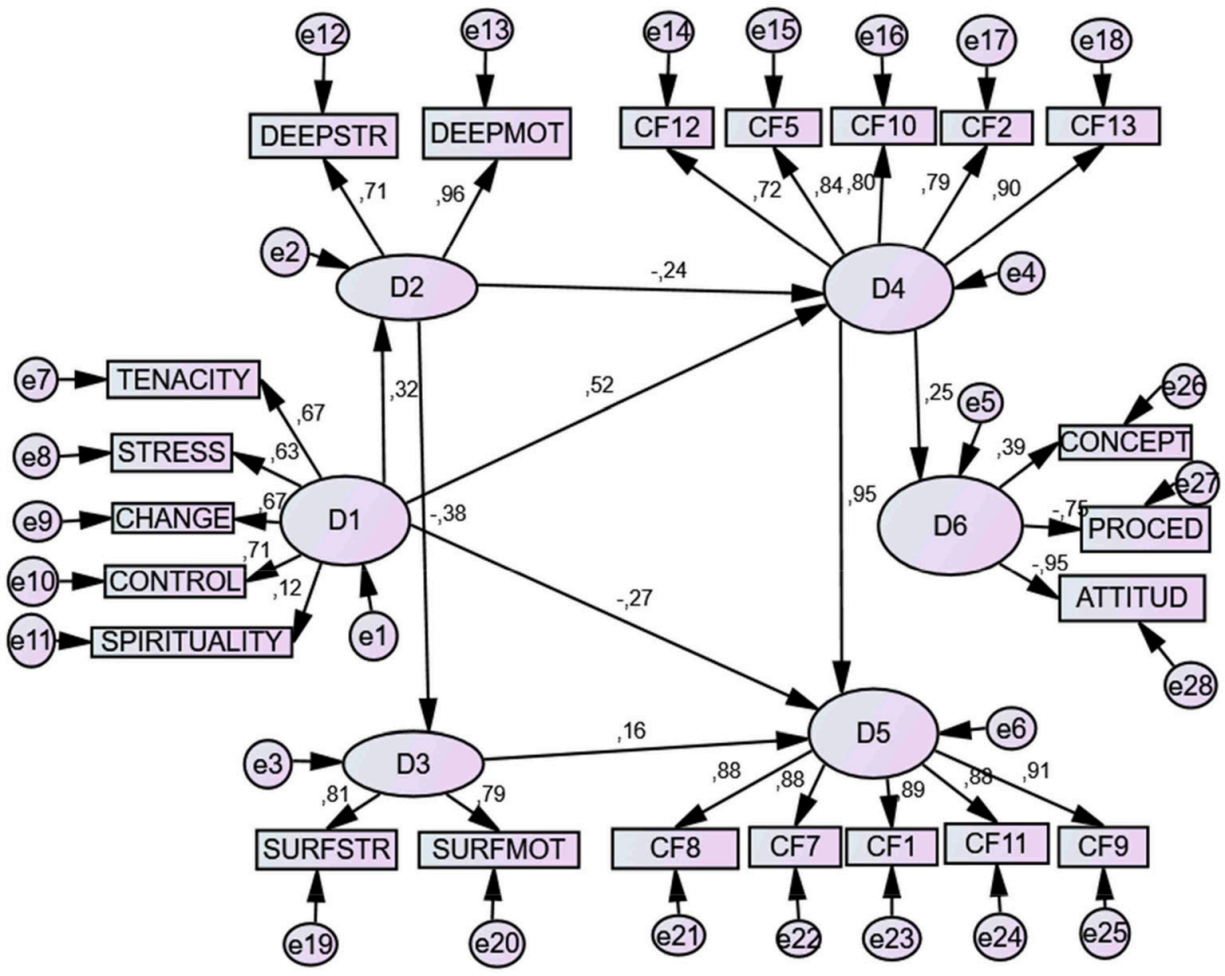
Structural model of relations proposed between Resilience (D1), Deep approach (D2), Surface approach (D3), Coping strategies centred on the problem (D4), Coping strategies centred in emotion (D5), and academic achievement (D6). TENACITY, Tenacity; ESTRES, Tolerance to stress; CHANGE, Change; CONTROL, Perception of control; SPIRITUALITY, Spirituality; D2, Deep approach; DEEPSTR, Deep strategy; DEEPMOT, Deep motivation; SURFSTR, Surface strategies; SURFMOT, Surface motivation; CF12, Communication of feelings and social support; CF5, Self-instructions; CF10, Positive re-evaluation and firmness; CF2, Search for help and family advice familiar; CF13, Search for alternative reinforcement CF8, Preparing for the worst; CF7, Reduction of anxiety and avoidance; CF1, Evasive distraction; CF11, Resigned acceptance; CF9, Emotional discharge and isolation. CONCEPT, Conceptual achievement; PROCED, Procedural achievement; ATT, Attitudinal achievement.

The final model is graphically represented in Figure 1.

## Discussion and conclusions

### Association relationship between resilience and learning approaches

As already predicted in *Hypothesis 1* (association) the relationship between *resilience* and *deep approach* appeared significant and positive, with a lower statistical weight of *spirituality*. This result is consistent with previous research, although it should not be forgotten that this factor has appeared as negatively associated with exhaustion, typical of burnout (de la Fuente et al., 2014). A plausible explanation is the fact that the sample comes from the Public Education System and this educational context, unlike the private religious system, has a resilience profile in which this factor does not seem a constituent of resilience - at least in the format evaluated by the CD-RISC used - as previous research has already shown (González-Torres and Artuch-Garde, 2014). In the case of the *surface approach* of learning, the expected general negative relationship did not appear, while the tenacity factor did, which would indicate the low level of persistence in the task of students with a surface learning approach. However, this expected relationship does appear as a negative indirect effect in the structural model (hypothesis 4). This novel aspect is important because it would contribute meta-motivational elements to the meta-cognitive ones of the mentioned construct. Thus, while students with a deep focus are also tenacious, have a perception of control, manage stress well and adapt to change, students with a surface focus do not have these characteristics and, moreover, are not tenacious. Previous evidence has corroborated some similar evidence of this pernicious relationship between *surface approach* and negative emotionality (Esquivel et al., 2009). In addition, there is ample evidence of the importance of this personal trait for learning and academic achievement, so that the absence of tenacity would be a negative predictor of commitment to learning and positive for burnout (de la Fuente et al., 2014). These results would confirm the importance of the relationship between meta-motivational (resilience) and meta-cognitive variables (learning approaches), already proposed in the CLSPS™ model, since previous works have reported the relationship between academic confidence and the deep learning approach (de la Fuente et al., 2013b).

### Association relationship between resilience and coping strategies

Hypothesis 2 (association), which analyses the relationships between resilience and coping strategies, was fulfilled, since there was a positive correlation between the *resilience* and *problem-focused coping strategies*, and negative between *resilience and emotion-focused coping strategies*, with a negative relationship with focused strategies in the cut emotions (evasive distraction) or inhibition (emotional discharge and isolation, resigned acceptance and preparing for the worst). Specifically, the behavioral components of resilience—especially *tenacity, change management, and perceived control*—are those that have appeared associated with behaviors of *self-instruction, positive reevaluations* and *firmness*. Therefore, the *resilience* of university students is confirmed, and is positively associated with problem-coping strategies. This finding shows the behavioral support of coping strategies (as meta-emotional variable) of resilience (as meta-emotional variable), providing empirical evidence for the relationship. In fact, few studies have reported on the relationship between resilience and coping strategies in the university population (Orozco, 2007; Li, 2008; de la Fuente et al., 2013a; Terzy, 2013).

These results are in line with previous research, which has shown a similar negative relationship between *personal self-regulation* and *coping strategies*, especially in those focused on emotion (de la Fuente et al., 2013a). Previous research has also shown with some clarity the relationship between *resilience and personal self-regulation*, and they are in line with these results (González-Torres and Artuch-Garde, 2014). The students who achieved medium-high scores in global resilience (Rodríguez and Valdivieso, 2008) also did well in aspects such as *the perception of control* (believing that they controlled the situation) and *change* (related to the possibility of establishing relationships on which to lean and to be flexible to adapt to new situations). These students also stood out for using strategies that focused on the problem. These aspects are positive to foster the search for solutions and the acquisition of the responsibility to solve problems, developing as autonomous people and being aware of the situation of stress to which they are exposed.

A relevant aspect worthy of mention in this research is that the results provide empirical evidence of how coping strategies are inherent in resilient conduct, which highlights the protective mechanism of resilience in health. Much recent evidence shows the association between resilience and health (Vinaccia et al., 2012; Villasana et al., 2016), but the mediating role of coping strategies as conduct associated with or inherent in resilience has not been sufficiently established.

### Association relationship between learning approaches and coping strategies

Hypothesis 3 (association) was partially verified in the same way, since there was a negative relation of the *deep approach* with the strategies focused on the emotion, but the expected effect on the positive relation did not appear with some strategies focused on the problem (self-instruction, positive reassessment and firmness). This scant use of the students' emotional coping strategies with the deep approach can be explained by their low experience of stress and high level of self-regulation, which would mean that university students with these characteristics would not need to apply them. In a previous work, it has been found that a deep learning approach is accompanied by greater planning, and more self-regulating behavior and, therefore, on those strategies that are more focused on the problem. The previous data also verify the existence of a negative relation between the surface approach and the self-regulated learning (de la Fuente et al., 2017b).

### Structural prediction of academic performance

Hypothesis 4 (structural) was validated because the results show that the final model had acceptable values, with statistically significant different indicators to default model (Chi-Square and Degrees of freedom). Values higher than.90 in the NFI (Normed Fix Index), RFI (Relative Fit Index), IFI (Incremental Fit Index), TLI (Tucker–Lewis index), CFI (Comparative Fit Index), HOELTER (Hoelter Index) > 200, and RMSEA (Root Mean Square Error of Approximation) = 0.08 index, indicate a good fit of the model (Byrne, 1989; Bentler, 1990).

The latent variable *resilience* and components of have a significant linear and predictive positive relationship to the *deep approach* (direct effect) and a negative one to the *surface approach* (indirect effect) as well as being a negative predictor of *problem-focused strategies* (direct effect) and negative to those *emotion-focused strategies* (indirect effect). Also, *resilience* had a positive linear predictor relationship, in conjunction with the *deep approach* (indirect effect) and *strategies focused on the problem of academic achievement* (direct effect). This relationship has appeared with differential effects for conceptual, procedural and attitudinal performance. There is previous evidence of partial performance-predictive relationships (Dwyer and Cummings, 2001). Zapata (2013) reported the positive predictive relationships of the deep approach to conceptual achievement, and personal self-regulation to procedural and attitudinal achievement in university students.

## Conclusions and implications

### Conclusions

In conclusion, these results establish the associative and predictive multidirectionality of the different variables in predicting university academic performance. Indeed, *resilience* (meta-motivational variable) predicts the type of learning approach (meta-cognitive variable) and the type of coping strategies (meta-emotional variable), and all three predict jointly academic performance and multidirectionality. This directionality is novel in that it empirically establishes the effect of the meta-motivational *attitudinal* level of learning (resilience) at the *procedural* meta-emotional level (coping strategies) and meta-cognitive level (learning approaches). Until now directionality was one-way or inverse in favor of pre-eminent cognitive variables, such as learning approaches or other meta-cognitive variables, as determinants of theoretical models of university learning (Biggs and Tang, 2012). Moreover, this empirical validation has entailed an advance on previous research, being based on more robust linear structural methodologies, especially in relation to the value of resilience and its relation to academic stress (Hartley, 2011) through coping strategies (Leipold and Greeve, 2009; Soucy et al., 2011; McLafferty et al., 2012).

However, this research also presents *limitations*, since it must be borne in mind that the variables with which we are working are of a personal nature and how the students acquired the previous learning with which they arrive at the university is unknown, as is the context of the stressful situations to which they have been exposed in their learning history. Nor has the role of the *gender* variable been considered in this model, which previous research has proved to have an effect (Stöber, 2004; Rubin et al., 2016). For future research, some of these instruments should be revalidated to ensure structural adequacy and factorial invariance as a preliminary step to their use, given that some inconsistencies have been found in the results, such as the low reliability of the Scale of Spirituality (Resilience) in this university sample.

### Implications

At the *research* level, an important implication is related to the necessary update of meta-motivational and meta-emotional variables in university learning processes as an important correlate of meta-cognitive processes (Biggs et al., 2001). For this purpose and the analysis of multiple relationships, the CLSPS™ model can be considered a relevant heuristic.

At the level of *applied psycho-educational practice*, in view of the results presented, a first relevant implication is the importance of evaluating and intervening in these variables for use during the counseling and health orientation processes in the university psycho-educational services (Hamdan-Mansour et al., 2009; Bartley et al., 2010; Hamaideh, 2011; Regehr et al., 2013). The evaluation of resilience and coping strategies could be helpful in selecting intervention programs to alleviate negative emotionality (and, if applicable, burnout) among university students, even using mindfulness interventions (Hoge et al., 2007; Caldwell et al., 2010; Lorenz et al., 2010). It is also imperative to consider interventions for the improvement of misaligned coping strategies, characteristic of students' differing learning approaches (deep vs. surface). In addition, another important implication is the need to use on-line screening tools for a first approach and help to university students. The *e-Coping with Academic Stress* (de la Fuente et al., 2015) tool allows self-evaluation and improvement of these factors among university students and opponents. It is important to move toward technological developments of this type.

### Future research directions

It is essential to advance in the relationships that these variables maintain with others, such as academic emotions or inadequate strategies of stress management, through substance use and other behavior harmful to health in university students (Chou et al., 2011; Bhullar et al., 2014). It is also important to analyse perceived inconsistencies in hypothetical relationships. In addition, the analyzed variables should be inserted into current learning strategies models, due to the potential of their contribution to knowledge of the role of meta-motivational and meta-affective strategies during university learning and health problems, produced by a maladjusted way of facing academic demands (Fernández-González et al., 2015; Freire et al., 2016).

## Ethics statement

All subjects gave written informed consent in accordance with the Declaration of Helsinki. The protocol was approved by the “BIOETIC RESEARCH COMMITE” OF UNIVERSITY OF ALMERIA. Human data were collected according to the Code of Practice of the Council of Psychology of Spain and were kept according to the Spanish Data Protection Act.

## Author contributions

Jd: Coordination of R & D Project; Data collect; Data analysis; Final writing. MF: Final writing; Analysis of data. MC: Review research; Data collect. MV: Data collect. MG: Review research; Final writing. RA: Review research; Analysis of data.

### Conflict of interest statement

The authors declare that the research was conducted in the absence of any commercial or financial relationships that could be construed as a potential conflict of interest.
